# Reliability of the Multiplex CytoBead CeliAK Immunoassay to Assess Anti-tTG IgA for Celiac Disease Screening

**DOI:** 10.3389/fmed.2021.731067

**Published:** 2021-09-21

**Authors:** Diyora Abdukhakimova, Kuanysh Dossybayeva, Anna Grechka, Zhaina Almukhamedova, Alyona Boltanova, Larissa Kozina, Kadisha Nurgaliyeva, Liliya Hasanova, Matthew N. Tanko, Dimitri Poddighe

**Affiliations:** ^1^School of Medicine, Nazarbayev University, Nur-Sultan, Kazakhstan; ^2^Clinical Academic Department of Pediatrics, National Research Center for Maternal and Child Health, University Medical Center, Nur-Sultan, Kazakhstan; ^3^Department of Clinical Diagnostic Laboratory, National Scientific Medical Center, Nur-Sultan, Kazakhstan; ^4^Clinical Academic Department of Laboratory Medicine, Republican Diagnostic Center, University Medical Center, Nur-Sultan, Kazakhstan

**Keywords:** celiac disease, children, anti-tissue transglutaminase antibody, ELISA, multiplex assay, screening, immunoglobulin A

## Abstract

**Background and Objective:** The diagnosis of Celiac Disease (CD) is first based on the positivity for specific serological markers. The CytoBead CeliAK immunoassay simultaneously measures antibodies (IgA) directed to tissue transglutaminase (tTG), endomysium (EMA), and deamidated gliadin (DG), in addition to providing a control for total IgA levels. The aim of this study is to assess the reliability of this multiplex assay to detect anti-tTG IgA positive patients, compared with a conventional single-parameter enzyme-linked immunosorbent assay (ELISA).

**Methods:** Serum samples from 149 pediatric patients were assessed by both CytoBead CeliAK immunoassay and ELISA, in order to evaluate their concordance for the measurement of anti-tTG IgA.

**Results:** The measurement of anti-tTG IgA by CytoBead CeliAK immunoassay basically showed a complete concordance rate with the conventional and single-parameter ELISA, according to the respective cutoff values (3 U/ml and 10 U/ml).

**Conclusions:** Our comparative analysis demonstrates a substantial equivalency between multiplex CytoBead CeliAK assay and the single-parameter conventional ELISA to assess anti-tTG IgA antibody in the context of the screening for CD in children. Importantly, CytoBead CeliAK assay could present some preanalytic, analytic, and economic advantages.

## Introduction

Celiac disease (CD) is a systemic immune-mediated disorder characterized by a very variable clinical expression, which ranges from classical symptoms of malabsorption to mild gastrointestinal complaints, passing through a multitude of different extra-gastrointestinal manifestations, that can be isolated or combined with the former (1). Importantly, CD in children can be even asymptomatic and, in these cases, can be investigated only later during adolescence or adulthood, because of some long-term complications, such as growth impairment, pubertal disorders, bone density reduction, fertility issues, occurrence of intestinal malignancy, and/or other autoimmune disorders (2, 3). Considering the significant prevalence of CD in children (around or at least 1% of the general population) and its raising incidence (4), CD should be actively sought, especially in those countries where the dietary regimens have been changing (with an even greater presence of wheat foods) and/or this diagnosis has not been appropriately considered until recent years (5–7).

The diagnosis of CD is defined by the demonstration of atrophic (small bowel) enteropathy in individuals exposed to gluten-based foods, which represent the necessary environmental disease trigger. However, the diagnostic workup starts with serological investigations mainly assessing the presence of anti-tissue transglutaminase antibody (anti-tTG) and/or anti-endomysium antibody (EMA), even though other markers may be variably used (e.g., anti-deamidated gliadin peptides, anti-gliadin antibody) (8). In detail, anti-tTG IgA performed by enzyme-linked immunosorbent assay (ELISA) resulted to be the most accurate marker to predict CD: its sensitivity and specificity are generally considered >90–95% (9, 10). Moreover, their role in the diagnostic workup of CD in children was upgraded from screening to diagnostic test able to “replace” the histologic confirmation under some specific conditions, according to the ESPGHAN guidelines. Indeed, provided that the value of anti-tTG IgA is 10 times greater than the upper limit of the reference range, symptomatic children who are also EMA positive and carriers of CD predisposing HLA-DQ genotypes (HLA-DQ2 and HLADQ8), can receive a final diagnosis of CD without any invasive procedure (duodenal biopsy) (11).

Therefore, the assessment of anti-tTG IgA is currently considered a “mandatory” and initial step for CD screening (and, in general, its final diagnosis), but other serological markers can be variably useful in the diagnostic workup of CD. In our pediatric center, we started using the novel multiplex CytoBead CeliAK diagnostic kit, which concomitantly assesses anti-tTG IgA, EMA, and anti-deamidated gliadin (anti-DG) antibody, in addition to providing a qualitative control for total serum IgA. However, before replacing the conventional ELISA for anti-tTG IgA, it is important to have evidence that the assessment of this antibody with this new multiplex assay is as much reliable.

In this preliminary study, we aimed to assess the reliability of the multiplex CytoBead CeliAK diagnostic kit as regards the identification and measurement of anti-tTG IgA, compared to the conventional single-parameter ELISA.

## Materials and Methods

### Study Design

This cross-sectional study investigated the CD serology in 149 pediatric patients admitted to the National Research Center for Maternal and Child Health (NRCMCH) of the University Medical Center (UMC), affiliated with the Nazarbayev University School of Medicine (NUSOM) in Nur-Sultan (Kazakhstan). The study period comprised between July and November 2020.

The study was approved by both the Institutional Research Ethical Committee of the Nazarbayev University (application n. 205/28112019, approved on January 23rd, 2020) and the Institutional Review Board of UMC (decision n.2.1, approved on December 19th, 2019). Patients' guardians provided signed informed consent for the participation in this study.

### Study Population and Sample Preparation

The study participants were all pediatric patients (age range: 2–17 years) followed in the Programs of Pediatric Gastroenterology, Pediatric Endocrinology and Pediatric Rheumatology of the Clinical Academic Department of Pediatrics at the NRCMCH. All children whose guardians accepted to participate in this research were included in this study. Therefore, the exclusion criteria are as follows: a previous or concomitant diagnosis of primary immunodeficiency, the administration of intravenous immunoglobulin in the previous 6 months, and the use of anti-B-cell biological therapy (e.g., rituximab). After obtaining the guardians' informed consent, the available secondary data about patients' demographic and clinical characteristics were retrieved from clinical records, and a small amount of blood was collected during the usual medical workup. The serum was then obtained by centrifugation, divided in aliquots, and frozen for preanalytic storage at −80°C.

### CD Screening by Multiplex CytoBead CeliAK

The National Scientific Medical Center (NSMC, Nur-Sultan, Kazakhstan) analyzed the CD serology by using the CytoBead CeliAK kit and the automated AKLIDES® equipment (Medipan GmbH, Blankenfelde-Mahlow, Germany). This multiplex assay can simultaneously assess anti-tissue transglutaminase IgA (anti-tTG IgA), IgA against endomysium (EMA), and anti-deamidated gliadin (anti-DG) antibody, in addition to providing a qualitative control for total serum IgA (IgA deficient if <500 AU/ml). All these tests are performed by using a single slide including different wells, each one specific for one analytical parameter, as summarized below.

This methodic is based on indirect immunofluorescence assay for the quantitative determination of anti-tTG IgA (n.v. 0–3 U/ml) and anti-DG (n.v. 0–6 U/ml) in human serum by using antigen-coated (human tTG and DG, respectively) beads. Tissue sections of monkey esophagus in the same kit allow the semiquantitative determination of EMA, based on indirect immunofluorescence method. The CytoBead CeliAK immunoassay (GA Generic Assays GmbH) uses a combination of monkey-esophagus cryostat tissue sections and autoantigen-coated fluorescent microbeads (Red 550, excitation 610 nm and emission 690 nm; sizes 9 and 15 μm; PolyAn GmbH, Berlin, Germany) on slides with compartmented wells for simultaneous autoantibody analysis, as described elsewhere in detail (12). Briefly, patients' samples at a dilution of 1:10 were incubated for 30 min at room temperature in each well. Unbound serum components were removed by a subsequent wash cycle. The second incubation of anti-human IgA conjugated to fluorescein isothiocyanate (FITC) (Seramun Diagnostica, Heidesee, Germany) for 30 min at room temperature in darkness was followed by another wash cycle to remove excess secondary antibody-conjugated molecules. The slides were washed five times in PBS and, thus, examined by automated fluorescence microscopy with the AKLIDES® system. In detail, the fluorescence microscope was equipped with a FITC filter (excitation 495 nm; emission 519 nm, EUROStar, Euroimmun AG, Lübeck, Germany), and the slides underwent automated analysis through the digital imaging platform AKLIDES® (12, 13).

### Anti-tTG IgA Screening by ELISA

The ELISA to measure anti-tTG IgA levels was performed at the Republican Diagnostic Center (RDC) of the University Medical Center (UMC) in Nur-Sultan, Kazakhstan.

The following kit was used: anti-tissue transglutaminase IgA (ORGENTEC Diagnostika GmbH, Mainz, Germany). This analysis was performed by a fully automated procedure through the diagnostic equipment Alegria® (ORGENTEC Diagnostika GmbH, Germany), by using 10 μl of undiluted sample. The normal range for anti-tTG IgA was 0–10 U/ml.

## Results

### Demographic and Clinical Characteristics of the Study Population

Overall, 149 children (female, *n* = 96, 64%; male, *n* = 53, 36%; age, 10.7 ± 4.2 years) were enrolled in this study and, thus, underwent the serological screening for CD.

Our center is a tertiary pediatric hospital admitting patients affected with chronic disorders. The Programs of Pediatric Gastroenterology, Pediatric Rheumatology and Pediatric Endocrinology participated in this study. Therefore, all enrolled children were affected/suspected and/or already diagnosed with one or more diseases included in the profile of these pediatric subspecialties. The detailed description of the clinical background of this cohort of patients is summarized in Table 1. The most common diagnoses were juvenile idiopathic arthritis (58%), type 1 diabetes mellitus (14%), and juvenile scleroderma (8%).

**Table 1:** Clinical characteristics of patients.

**Clinical diagnosis**	**Female**	**Male**	**Total**
Acute rheumatic fever	–	1	1
Behcet's disease	2	–	2
Thyrotoxicosis	1	–	1
Ulcerative chronic colitis	1	2	3
Chronic diarrhea	3	1	4
Crohn's disease	1	–	1
Diffuse toxic goiter	–	1	1
Gastritis	–	1	1
Hypopituitarism	–	1	1
Juvenile dermatomyositis	3	–	3
Juvenile systemic sclerosis	1	–	1
Diseases of the biliary tract	–	1	1
Short stature	1	2	3
Systemic connective tissue disease	1	–	1
Systemic lupus erythematosus	5	1	6
Juvenile scleroderma	7	5	12
Type 1 diabetes mellitus	12	9	21
Juvenile idiopathic arthritis	58	28	86
**Total**	**96**	**53**	**149**

### Serological Screening by Multiplex CytoBead CeliAK Immunoassay

All these 149 children were screened for CD by using the CytoBead CeliAK immunoassay. Among them, 10 children were positive for anti-tTG IgA, and they also resulted to be all positive for EMA. Additionally, five patients were EMA positive but anti-tTG IgA negative. As regards anti-DG IgA, six patients resulted positive: interestingly, all these patients were EMA positive, but only one was also anti-tTG IgA positive. In five patients, the diagnostic kit evidenced low IgA levels, indicating that the CD serological test cannot be reliable. The detailed serological profiles of these 15 patients who showed positivity in at least one of the parameters assessed by this diagnostic kit, are shown in Table 2.

**Table 2:** CD serological panel of positive patients by CytoBead CeliAK immunoassay (columns 3-4-5) and comparison with the ELISA results (columns 6).

**Pt**.	**Current**	**Anti-tTG IgA [CeliAK]**	**EMA [CeliAK]**	**Anti-DG IgA [CeliAK]**	**Anti-tTG IgA [ELISA]**
	**diagnosis**	**(0-3.0 U/ml)**	**(Pos/Neg)**	**(0-6.0 U/ml)**	**(0-10 U/ml)**
1	IBD?	0.6	**Positive**	**34.9**	0.3
2	IBD?	0.3	**Positive**	**14.7**	0.3
3	JIA	0.3	**Positive**	**7.4**	0.5
4	JIA	0	**Positive**	**6.3**	0.4
5	JIA	0	**Positive**	**10.6**	3.4
6	JIA	**103**	**Positive**	1.6	**89.3**
7	JIA	**31.2**	**Positive**	0.06	**45.4**
8	C. diarrhea	**102.2**	**Positive**	2.3	**27.7**
9	C. diarrhea	**100.6**	**Positive**	**102.6**	**200**
10	JS	**5.9**	**Positive**	0.9	**10.4**
11	T1DM	**45.9**	**Positive**	0.1	**25.4**
12	T1DM	**75.3**	**Positive**	2.7	**21.9**
13	T1DM	**5.4**	**Positive**	3.1	7.2
14	T1DM	**100.6**	**Positive**	1.9	**200**
15	T1DM	**38**	**Positive**	0.9	**44**

*IBD, inflammatory bowel disease; JIA, juvenile idiopathic arthritis; C., chronic; JS, juvenile scleroderma; T1DM, type 1 diabetes mellitus. Bold values mean “positive” values, higher than the reference values for the respective parameter*.

At the moment, only four (out of these 15 patients) could undergo upper gastrointestinal endoscopy with duodenal biopsy: the diagnosis of CD was histologically confirmed in all these four patients according to Marsh classification (pt.2, grade 3B; pt. 6, grade 3A; pt. 9: grade 3A; pt. 15: grade 3A).

### Anti-tTG IgA Serological Screening by Conventional ELISA

**Figure 1:**
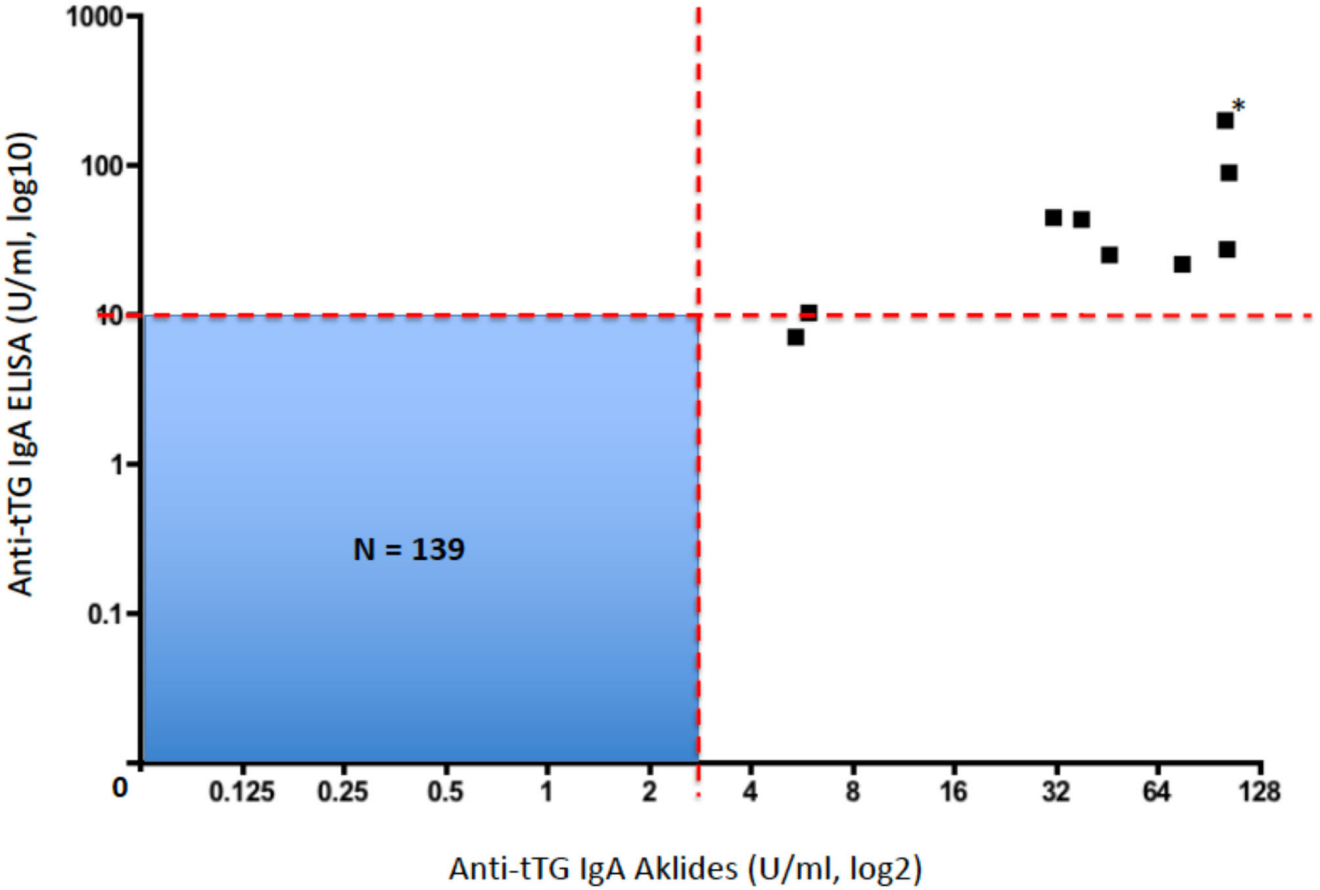
Plot correlating anti-tTG IgA levels by CytoBead CeliAK immunoassay and anti-tTG IgA levels by ELISA (*this black square corresponds to two patients with identical values; *N*, total number of patients included in this blue quadrant; red dashed line, respective cutoff values for each assay).

All these 149 children were also screened for CD by using a conventional ELISA measuring anti-tTG IgA. All those 10 patients who resulted anti-tTG IgA positive by CytoBead CeliAK immunoassay, also showed elevated anti-tTG IgA by the single-parameter ELISA. In detail, as showed in Table 2, nine patients resulted as anti-tTG IgA positive by ELISA according to the conventional cutoff (10 U/ml), and only one patient showed elevated anti-tTG IgA (7.2 U/ml) but below the cutoff. Importantly, no patients being anti-tTG IgA negative by CytoBead CeliAK immunoassay, showed positive results through ELISA, as graphically shown in Figure 1.

Therefore, the anti-tTG IgA level measured by CytoBead CeliAK immunoassay basically showed a complete concordance rate with the conventional single-parameter ELISA for anti-tTG IgA. As said, 9 out of 10 patients who were anti-tTG IgA positive by CytoBead CeliAK immunoassay were confirmed as positive by ELISA as well. However, in the remaining positive patient by CytoBead CeliAK immunoassay (anti-tTG IgA = 5.4 U/ml, n.v. = 0–3 U/ml) who showed anti-tTG IgA level below the conventional cutoff by ELISA (7.2 U/ml, n.v. = 0–10 U/ml), this latter value was in the upper normal range: indeed, such a value (7.2 U/ml) was >2 times greater than the highest value (3.4 U/ml) and much higher than the mean value (0.34 ± 0.58 U/ml) observed among the 139 children negative for anti-tTG IgA antibody by CytoBead CeliAK immunoassay. Moreover, this patient was EMA positive.

## Discussion

In this study, we assessed the reliability of the new CytoBead CeliAK immunoassay to assess anti-tTG IgA antibodies for the screening of CD in children, in comparison with a conventional and single-parameter ELISA test. In this regard, we found that CytoBead CeliAK immunoassay performed as good as the ELISA: importantly, the former test showed almost 100% concordance rate with the latter one, in terms of negative and positive results, according to the respective cutoff values. Indeed, the CytoBead CeliAK assay correlated very well with anti-tTG IgA by ELISA, as shown in Figure 1.

It is well-known that anti-tTG IgA is the most specific and sensitive serological marker for CD (8–10), and such a very strong correlation and concordance between CytoBead CeliAK assay and the usual methodic (single-parameter ELISA) suggests that the former assay can be safely used in the CD screening process as well as the latter one. Indeed, as regards the assessment of anti-tTG IgA, this multiplex assay uses anti-human tTG-coated beads (12, 13), which significantly reduces the possibility of having false-positive results, as reported for some initial enzyme-linked immunosorbent assays (14, 15). This aspect is also important considering the wide range of autoantibodies that may be observed in CD patients and, indeed, some of them are also affected with other autoimmune and/or rheumatic comorbidities (16–18).

Importantly, at a similar cost as the conventional ELISA, the CytoBead CeliAK assay has the advantage of simultaneously measuring several CD-related serological parameters (that are useful and/or required for the complete diagnostic workup of CD in children), in addition to other positive preanalytic and analytic aspects, as discussed below.

Indeed, the CytoBead CeliAK assay is performed by an automated procedure with AKLIDES® equipment, which enables the simultaneous and objective (not operator-dependent) assessment of CD-specific autoantibodies (anti-tTG IgA, EMA, and anti-DG IgA) (12). Thus, the comprehensive CD serology provided by this multiplex assay can significantly reduce the workload in the laboratory routine. Moreover, it concomitantly allows to identify those patients who are affected with IgA deficiency (indicated by the lack of a fluorescent signal from the well-coated with anti-human IgA microbeads), which is known to impair the reliability of any CD screening test based on IgA autoantibody. Indeed, it is a mandatory and good clinical practice to request the concomitant (and separate) assessment of total IgA in serum, whenever a patient is screened for CD with anti-tTG IgA by a conventional single-parameter ELISA (19, 20). Finally, in addition to this qualitative assessment of total IgA levels, as explained, CytoBead CeliAK assay provides information on other CD-related antibodies (EMA and anti-DG IgA), which usually would require separate analyses and additional workload, if done by using different single-parameter tests. These different antibodies may also help to define prognostic aspects of CD in different patients, even though at the moment only anti-actin IgA showed a correlation with villous atrophy, in addition to anti-tTG IgA (21, 22).

All these analytic aspects reflect on the economical side as well. Indeed, in addition to being more friendly for the routine laboratory practice, the CytoBead CeliAK assay could be more cost-effective in the screening and complete diagnostic workup for CD. Indeed, considering all the costs for consumables, reagents, multiplex diagnostic kit, and human work, we spent around 7,000 KZT (currently corresponding to US$15–16) per patient, which then included anti-tTG, EMA, and anti-DG autoantibodies, in addition to the assessment of total IgA serum levels. In Kazakhstan, only the cost to perform the ELISA for anti-tTG IgA was 5,250 KZT. The additional costs to perform EMA and total serum IgA analysis would be 12,600 and 2,200 KZT, respectively. Therefore, the same CD serology panel tested with a multiplex technology by CytoBead CeliAK assay (costing around 7,000 KZT, as said above) would cost around 20,000 KZT (currently corresponding to US$47) by using the conventional single-parameter assays (without considering the anti-DG assay), resulting thus almost three times less expensive in Kazakhstan. Indeed, in this country (based on the current health system organization), the economic factor has a significant impact on the diagnostic workup and, consequently, diagnostic rate of CD (23), which is likely to be still underdiagnosed based on our recent analyses of the CD-predisposing genetic background in this population and, in general, in Central Asia (24, 25). However, the costs of screening test and diagnostic workup for CD is also relevant in developed countries, as discussed by several authors (26–28). Finally, if we consider children as a target population for the CD screening, the multiplex analysis by CytoBead CeliAK assay requires only one sample (thus, a lesser amount) of blood, rather than several collection tubes, which may be an issue especially in infants and younger children.

Importantly, in addition to the CD screening in patients affected with specific extra-gastrointestinal diseases or manifestations (3, 29, 30), the availability of a cost-effective, but reliable, serological test may be considered for large scale or mass screening, the debate of which is still open (5, 31, 32).

To conclude, it is important to disclose the several limitations of our present research. In addition to the small sample size and inclusion of pediatric patients only, we could not perform the same reliability analysis and comparison for EMA, due to our budget constraint and the current unavailability (in our laboratories) of the reagents and diagnostic kits to perform EMA analysis by the conventional single-parameter indirect immunofluorescence assay. Finally, the current limitations in the organization and access of the healthcare system in the country did not allow us to perform the histological examination of duodenal mucosa in the short term and to all the serologically positive patients. Therefore, additional, larger, and more complete studies are needed to confirm our preliminary and initial results with CytoBead celAK immunoassay.

## Conclusion

Our comparative analysis preliminarily demonstrates a substantial concordance rate between multiplex CytoBead CeliAK assay and the single-parameter conventional ELISA for assessing the anti-tTG IgA antibody in the context of the screening for CD in children. Importantly, CytoBead CeliAK assay could have some preanalytic, analytic, and economic advantages, which potentially make it a cost-effective method to be used in the screening and diagnostic workup of CD on a large-scale testing.

## Data Availability Statement

The raw data supporting the conclusions of this article will be made available by the authors, without undue reservation.

## Ethics Statement

The studies involving human participants were reviewed and approved by Institutional Research Ethical Committee of the Nazarbayev University (application n. 205/28112019, approved on January 23rd, 2020) and the Institutional Review Board of UMC (decision n.2.1, approved on December 19th, 2019). Written informed consent to participate in this study was provided by the participants' legal guardian/next of kin.

## Author Contributions

DP and DA contributed conceptualization of the study and reviewed and edited the manuscript. AB, LK, KN, and MT contributed to the methodology used in this study. DA and KD performed formal analysis and carried out data curation. KD, AG, and ZA contributed to the investigation. DP, DA, and KD supervised the study and wrote the original draft of the manuscript. DP contributed to the funding acquisition. All authors have read and agreed to the published version of the manuscript.

## Funding

This research was funded by the Nazarbayev University, Grant Number 240919FD3912.

## Conflict of Interest

The authors declare that the research was conducted in the absence of any commercial or financial relationships that could be construed as a potential conflict of interest. Moreover, the funders had no role in the design of the study; in the collection, analyses, or interpretation of data; in the writing of the manuscript; or in the decision to publish the results.

## Publisher's Note

All claims expressed in this article are solely those of the authors and do not necessarily represent those of their affiliated organizations, or those of the publisher, the editors and the reviewers. Any product that may be evaluated in this article, or claim that may be made by its manufacturer, is not guaranteed or endorsed by the publisher.
